# X-SPEAR: An Extensible Framework for Spatial Panel Evaluation and Refinement

**DOI:** 10.64898/2026.10.13.756865

**Published:** 2026-10-20

**Authors:** Rushil Patel, Bryant Duong, Veer Patel, Rachel Lyn-Sue, Kyungbin Oh, Yuyan Cheng

## Abstract

Targeted spatial transcriptomics platforms such as 10x Genomics Xenium measure a predefined panel of genes. A gene is usually detected in fewer cells in the spatial assay than in the single-cell RNA sequencing (scRNA-seq) reference used to choose it, by an amount that differs from gene to gene. We present eXtensible Spatial Panel Evaluation And Refinement (X- SPEAR), a framework for designing and checking Xenium gene panels. Its marker-selection method, Decision-rule Aggregation for Rare-cell Typing (DART), learns short Boolean gene rules and keeps several gene-disjoint rules per cell type. DART first thins the reference to per-gene detection rates measured in a previous experiment, which we call a Xenium prior, so no Xenium data from the target sample are needed before the panel is ordered. A Xenium prior helped most when it matched the target’s assay chemistry and species, even when it came from a different tissue, and a mismatched prior could lower accuracy. With such a prior, DART outperformed four marker-selection methods on held-out Xenium data from ovarian cancer, mouse brain and cervical cancer at matched panel size: by 0.04–0.10 macro-averaged *F*_1_ score (macro- *F*_1_ ) over the Wilcoxon rank-sum test, 0.04–0.08 over COSine similarity-based marker Gene identification (COSG), 0.10–0.17 over Necessary and Sufficient Forest (NS-Forest) and 0.04–0.08 over the probe-set selection method Spapros. It reached the Wilcoxon test’s best accuracy with 42–69% fewer genes. DART’s rules agree with the classifier that uses the panel and name recognisable marker programs for each cell type. X-SPEAR also checks the probes of any panel for predicted off-target transcripts that are expressed in other cell types. X-SPEAR is available as an open-source Python package.

